# Injectable Thermo-Sensitive Chitosan Hydrogel Containing CPT-11-Loaded EGFR-Targeted Graphene Oxide and SLP2 shRNA for Localized Drug/Gene Delivery in Glioblastoma Therapy

**DOI:** 10.3390/ijms21197111

**Published:** 2020-09-26

**Authors:** Yu-Jen Lu, Yu-Hsiang Lan, Chi-Cheng Chuang, Wan-Ting Lu, Li-Yang Chan, Peng-Wei Hsu, Jyh-Ping Chen

**Affiliations:** 1 School of Medicine, Chang Gung University, Kwei-San, Taoyuan 33302, Taiwan; alexlu0416@gmail.com (Y.-J.L.); orange7975@yahoo.com.tw (Y.-H.L.); 2 Department of Neurosurgery, Chang Gung Memorial Hospital, Linkou, Kwei-San, Taoyuan 33305, Taiwan; ccc2915@cgmh.org.tw (C.-C.C.); ns3096@cgmh.org.tw (P.-W.H.); 3 Department of Chemical and Materials and Materials Engineering, Chang Gung University, Kwei-San, Taoyuan 33302, Taiwan; s904140328@yahoo.com.tw; 4 Department of Surgery, Chang Gung Memorial Hospital, Linkou, Kwei-San, Taoyuan 33305, Taiwan; mangojoneschan@gmail.com; 5 Department of Plastic and Reconstructive Surgery and Craniofacial Research Center, Chang Gung Memorial Hospital, Linkou, Kwei-San, Taoyuan 33305, Taiwan; 6 Research Center for Food and Cosmetic Safety, Research Center for Chinese Herbal Medicine, College of Human Ecology, Chang Gung University of Science and Technology, Taoyuan 33302, Taiwan; 7 Department of Materials Engineering, Ming Chi University of Technology, Tai-Shan, New Taipei City 24301, Taiwan

**Keywords:** hydrogel, drug delivery, cancer therapy, nanomedicine, chitosan, graphene oxide, shRNA

## Abstract

In this study, we aimed to develop a multifunctional drug/gene delivery system for the treatment of glioblastoma multiforme by combining the ligand-mediated active targeting and the pH-triggered drug release features of graphene oxide (GO). Toward this end, we load irinotecan (CPT-11) to cetuximab (CET)-conjugated GO (GO-CET/CPT11) for pH-responsive drug release after endocytosis by epidermal growth factor receptor (EGFR) over-expressed U87 human glioblastoma cells. The ultimate injectable drug/gene delivery system was designed by co-entrapping stomatin-like protein 2 (SLP2) short hairpin RNA (shRNA) and GO-CET/CPT11 in thermosensitive chitosan-*g*-poly(*N*-isopropylacrylamide) (CPN) polymer solution, which offers a hydrogel depot for localized, sustained delivery of the therapeutics after the in situ formation of CPN@GO-CET/CPT11@shRNA hydrogel. An optimal drug formulation was achieved by considering both the loading efficiency and loading content of CPT-11 on GO-CET. A sustained and controlled release behavior was found for CPT-11 and shRNA from CPN hydrogel. Confocal microscopy analysis confirmed the intracellular trafficking for the targeted delivery of CPT-11 through interactions of CET with EGFR on the U87 cell surface. The efficient transfection of U87 using SLP2 shRNA was achieved using CPN as a delivery milieu, possibly by the formation of shRNA/CPN polyplex after hydrogel degradation. In vitro cell culture experiments confirmed cell apoptosis induced by CPT-11 released from acid organelles in the cytoplasm by flow cytometry, as well as reduced SLP2 protein expression and inhibited cell migration due to gene silencing. Finally, in vivo therapeutic efficacy was demonstrated using the xenograft of U87 tumor-bearing nude mice through non-invasive intratumoral delivery of CPN@GO-CET/CPT11@shRNA by injection. Overall, we have demonstrated the novelty of this thermosensitive hydrogel to be an excellent depot for the co-delivery of anticancer drugs and siRNA. The in situ forming hydrogel will not only provide extended drug release but also combine the advantages offered by the chitosan-based copolymer structure for siRNA delivery to broaden treatment modalities in cancer therapy.

## 1. Introduction

Glioblastoma multiforme (GBM) is the most aggressive and lethal brain tumor in adults. It is characterized by rapid proliferation, high infiltration capacity, and chemoresistance. It is also highly recurrent even through multimodal interventions, making it challenging to treat. The prognosis is often bleak, with a median overall survival ranging from 12 to 15 months and a 5-year survival of 6.8% [1,2]. Current standard treatment for GBM is maximal surgical resection, followed by chemotherapy and radiation [3].

Irinotecan (CPT-11), a chemotherapeutic agent sold under the brand name Camptosar^®^, can be used in treating GBM [4,5]. However, the major obstacle that hinders successful chemotherapy is its lack of selective targeting. Indeed, chemotherapeutic drugs may not be able to distinguish between normal and malignant cells, resulting in attacking healthy cells while killing cancer cells minimally [6]. Furthermore, multi-drug resistance and poor pharmacokinetic properties are other challenges that we are facing in cancer treatment. Therefore, innovative targeting strategies with enhanced cancer selectivity and minimal adverse effects are needed. Graphene oxide (GO) has been widely used as a nanocarrier for drug delivery with advantages such as large surface area, two-dimensional planar structure, chemical and mechanical stability, and biocompatibility [7]. In addition, pH-responsive drug release from drug-loaded GO will provide an efficient intracellular release of therapeutic drugs in acidic organelles after the endocytosis of drug-loaded GO by cancer cells [8]. Nevertheless, limitations still exist in nanocarrier-based drug delivery systems. Nanocarriers tend to undergo rapid elimination from the reticuloendothelial system due to their small size, in addition to lacking a “smart” selectivity for tumor tissues [9]. Therefore, the targeted delivery of anti-tumor drugs for cancer therapy is considered to be more effective than traditional chemotherapy [10]. Indeed, an active targeting strategy could increase the ability of nanocarriers to be specifically recognized by tumor cells through modifying the carrier surface with a ligand, such as a monoclonal antibody [11]. The epidermal growth factor receptor (EGFR) is over-expressed by most brain tumors, but not by normal tissues, which explains the reason why cetuximab (CET), the EGFR monoclonal antibody, was developed specifically for brain tumor targeting [12]. Thus, modifying GO nanocarriers with CET could enhance its intracellular uptake by cancer cells through ligand-mediated targeted drug delivery [13].

On the other hand, a single drug might not be able to efficiently eliminate all cancer cells, either due to the heterogenic nature of malignant tumors or the existence of malignant cells at the disparate cell division or growth stage [14]. Therefore, combination therapy with different molecular targets is a promising strategy to vanquish drug resistance, reduce the possibility of tumor metastasis, and improve treatment outcomes [15]. Gene therapy as a strategy to curb tumor growth and invasion by the insertion of new genes (DNA and RNA) into target cells is an emerging therapeutic strategy for the treatment of GBM [16]. As an efficient method to post-transcriptionally turn off the expression of specific proteins and transcription factors, RNA interference (RNAi) using small interfering RNA (siRNA) emerges as therapeutics for cancer therapy [17]. Combinatorial siRNA delivery to human primary GBM using cancer-selective nanoparticles was demonstrated to be effective for multimodal therapeutic for cancer [18]. However, some hurdles need to be overcome for delivery of the negatively charged naked siRNA, including their biological instability, off-target effects, inability to passively diffuse across the negatively charged cell membranes, and easy degradation by ribonucleases [19]. To overcome these limitations, a number of siRNA delivery systems, including liposomes, polyplexes, and nanoparticles have been developed for siRNA delivery [20]. Nonetheless, as nanocarriers for siRNA delivery could be easily dispersed in vivo to render local delivery to target sites difficult for a prolonged period of time, sustained localized siRNA delivery is more desirable to enhance its clinical applicability.

As a member of the highly conserved stomatin superfamily, stomatin-like protein 2 (STOML2 or SLP2) was found to be widely expressed in mammalian tissues and considered to be a major mitochondrial inner membrane protein. Recently, several studies have reported that SLP2 plays an oncogenic role in tumor occurrence and progression [21,22]. One study found that SLP2 is one of the 16 most up-regulated proteins in super invasive cancer cells, implying that SLP2 might play a role in cancer metastasis [23]. However, the molecular mechanisms of SLP2 in the progression and development of cancer remain largely unknown. Specifically, SLP2 was fond to be markedly upregulated in glioma cells and glioma specimens. The up-regulation of SLP2 was shown to be significantly correlated with the World Health Organization (WHO) histological grade of gliomas, while patients with higher SLP2 expression levels had an overall shorter survival time compared to patients with a lower expression of SLP2 [24]. It was also demonstrated that the silencing of SLP 2 gene expression could drastically reduce the migration and invasive ability of glioma cells by inhibiting the transcriptional activity of nuclear factor kappa-light-chain-enhancer of activated B cells (NF-κB) and repressing the expression levels of NF-κB target genes, including matrix metallopeptidase 9 [25]. Therefore, SLP2 plays an important role in human glioma progression and pathogenesis.

Hydrogels are outstanding candidates for drug/gene controlled-release delivery systems. Considering hydrogels for the local release of therapeutics, an injectable hydrogel formulation with in situ sol-to-gel transition, triggered externally under physiological conditions after injection using light exposure or temperature/pH change, will be preferred over preformed hydrogels considering non-invasive surgical intervention during delivery [26]. Specifically, thermo-responsive hydrogels are one of the most studied classes of stimuli-responsive polymer systems. The self-assembly of these polymers could lead to sol–gel phase transition when the temperature was increased to above the lower critical solution temperature in an aqueous solution of such polymers. For biomedical and pharmaceutical applications, the poly(*N*-isopropylacrylamide) (PNIPAM)-based polymer is one of the most popular thermosensitive polymers intended for such application, with its phase transition temperature being around 32 °C [27]. Taking advantage of this unique property, a drug/gene-loaded thermal-sensitive polymer solution could be non-invasively administrated peritumorally or intratumorally into a tumor at room temperature using a syringe needle. After thermally induced phase transition at body temperature, the formed hydrogel will provide a gel depot for controlled drug/gene release in accordance with the degradation rate of the formed hydrogel [14,28,29]. Interestingly, an injectable, thermosensitive hydrogel combined with drug-loaded nanoparticles was shown to provide better control of drug release and proved to be an efficient drug delivery system with sustained drug release around tumor tissues for peritumoral chemotherapy [30]. On the other hand, hydrogels also appear to be a reliable solution to deliver two or more therapeutic agents concurrently. Combination cancer therapy by incorporating anticancer drugs and genes in carriers has the advantages of protecting drug bioactivity, limiting unwanted toxicity, improving hydrophilicity, prolonging duration time, controlling the rate of drug release, and showing synergistic anticancer effects [31,32].

Considering the advantages offered by polysaccharides for the development of hydrogels, the chitosan-based hydrogels play a crucial role in the development of new biomaterials for use in drug/gene delivery for its biocompatible and biodegradable natures. In situ forming chitosan-based hydrogels are very attractive for the local delivery of drugs by showing the sustained release of drug to enhance the drug bioavailability and reduce systemic toxicity [33]. Moreover, chitosan has attracted significant attention for siRNA delivery owing to its cationic nature for easy complexation with siRNA [34]. Indeed, chitosan nanoparticles were suggested to be an effective drug/gene delivery system for the treatment of prostate cancer by the co-delivery of irinotecan and Snail siRNA [35]. Previously, we developed a comb-like thermo-responsive polymer chitosan-g-PNIPAM (CPN), using chitosan as the backbone for pendant PNIPAM group grafting, which was accomplished by conjugating PNIPAM-COOH to chitosan by amide bond linkages. This thermo-responsive copolymer was proved to be biocompatible as a milieu for cell culture and drug delivery [36,37]. We postulate that this copolymer will be an excellent candidate, as an in situ forming hydrogel depot for cancer therapy, by the co-delivery of anticancer drugs and siRNA. To provide extended drug release, entrapping drug-loaded GO within CPN hydrogel should improve the drug release behavior. To further broaden the treatment modalities offered by CPN, siRNA gene delivery could be combined with drug delivery through the advantages offered by this chitosan-based hydrogel. Considering gene delivery, although both siRNAs and short hairpin RNA (shRNA) expression plasmids are common methods that mimic Dicer cleavage for sequence-specific targeting mRNAs, siRNAs are susceptible to nuclease destruction and cannot penetrate the cell membrane because of their highly charged backbone. Therefore, we will construct and use SLP2 shRNA plasmids in this study for effective gene delivery.

**Figure 1:**
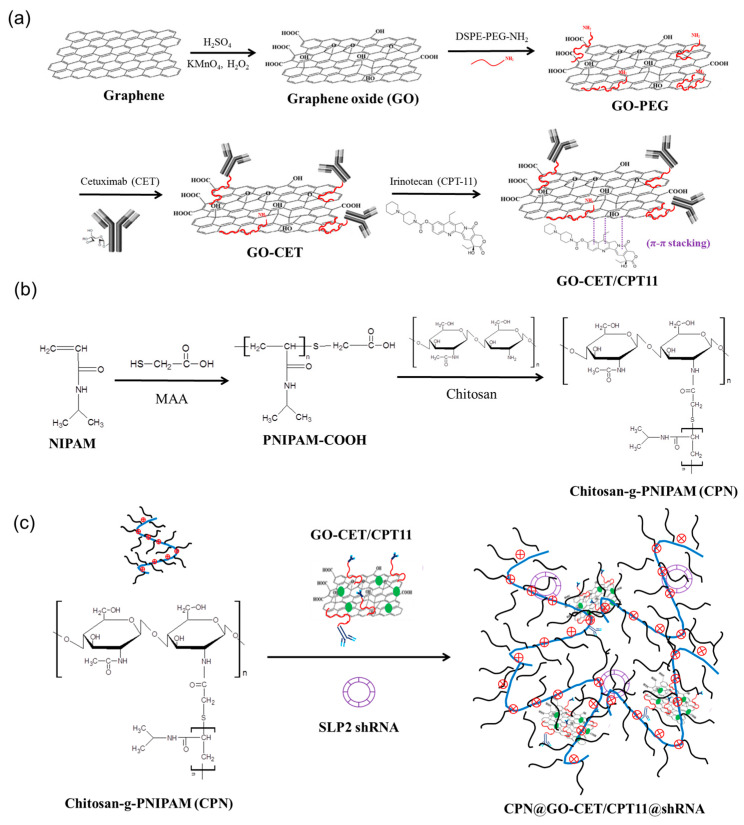
The flow diagram for synthesizing irinotecan (CPT-11) to cetuximab (CET)-conjugated graphene oxide (GO) (GO-CET/CPT11) for co-entrapment with stomatin-like protein 2 (SLP2) short hairpin RNA (shRNA) in chitosan-g-poly(*N*-isopropylacrylamide) (CPN). (**a**) GO produced by the modified Hummers method was modified with *N*-(aminopropyl polyethyleneglycol)carbamyl-distearoylphosphatidyl-ethanolamine 1,2-distearoyl-sn-glycero-3-phosphoethanolamine-*N*-[amino(polyethyleneglycol)] (DSPE-PEG-NH_2_) to prepare GO-PEG, followed by conjugation with CET to obtain GO-CET. CPT-11 was loaded onto GO-CET via π–π stacking interactions to form GO-CET/CPT11. (**b**) Chitosan-g-poly(*N*-isopropylacrylamide) (CPN) was synthesize by grafting carboxylic acid-ended poly(*N*-isopropylacrylamide) (PNIPAM) (PNIPAM-COOH), prepared by the free radical polymerization of *N*-isopropylacrylamide (NIPAM) and mercaptoacetic acid (MAA), onto chitosan backbone through amide bond linkages. (**c**) The GO-CET/CPT11 and SLP2 shRNA could be mixed with the thermosensitive CPN hydrogel at room temperature and entrapped within the polymer matrix after sol–gel transition in situ to form CPN@GO-CET/CPT11@shRNA.

Hence, in the present study, we aim to develop a localized, sustained co-delivery strategy of SLP2 shRNA and GO-CET/CPT11 (CPT-11 loaded to GO-CET) by utilizing the biodegradable CPN hydrogel as an injectable hydrogel depot for the treatment of glioblastoma in vitro and in vivo (Figure 1). The CPN hydrogel loaded with GO-CET/CPT11 or SLP2 shRNA was characterized during each preparation step as well as the drug/gene release characteristics. To study the synergistic anti-tumor efficacy of functionalized CPN hydrogel, U87 human glioblastoma cells were used to study cell cytotoxicity, SLP2 gene transfection, gene silencing, and cell apoptosis in vitro. Finally, by intratumoral injection of CPN hydrogel co-loaded with SLP2 shRNA and GO-CET/CPT11 (CPN@GO-CET/CPT11@shRNA) into nude mice bearing human glioblastoma U87 xenografts, the anti-tumor efficiency was also studied in vivo.

## 2. Results and Discussion

### 2.1. Synthesis and Characterization of GO, GO-PEG, GO-CET, and GO-CET/CPT11

**Figure 2:**
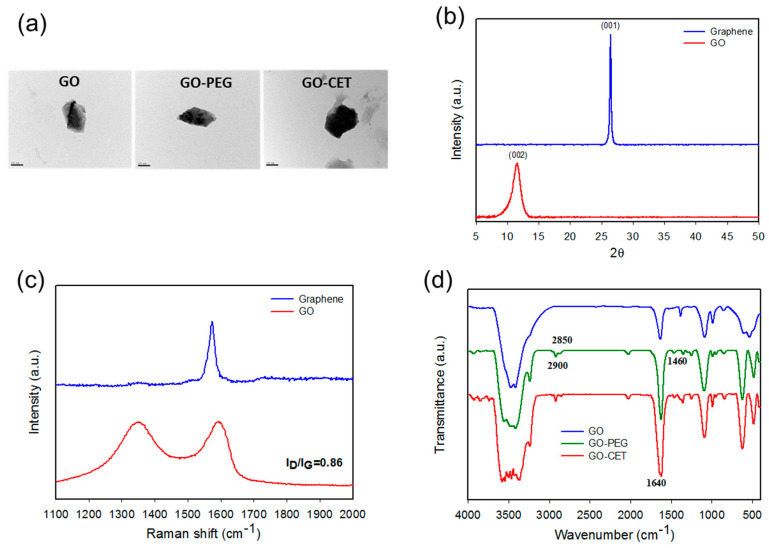
Characterization of nanocarriers by transmission electron microscopy (TEM) (bar = 100 nm) (**a**), X-ray diffraction (XRD) (**b**), Raman spectroscopy (**c**), and Fourier transform infrared (FTIR) spectroscopy (**d**).

The GO-based nanocarriers prepared as illustrated in Figure 1a were characterized after each synthesis step. From dynamic light scattering (DLS) analysis of particle size, the average particle diameters determined by DLS were 201 ± 1.5, 213 ± 0.8 nm, 250 ± 1.6 nm, and 279 ± 1.1 nm, for GO, GO-PEG, GO-CET, and GO-CET/CPT11, respectively, which could facilitate endocytosis by cancer cells [38]. The morphology of GO, GO-PEG, and GO-CET were observed under transmission election microscope (TEM). Figure 2a reveals the laminar stacking form of GO with ≈200 nm size, which is consistent with that from DLS analysis. After PEGylation and CET conjugation modification, GO-PEG and GO-CET show similar surface morphology with slightly larger size as expected. The GO exhibited a highly negative zeta potential value (−48.4 ± 0.59 mV) due to the presence of abundant oxygen-containing functional groups on its surface. The zeta potential changed to −34.4 ± 0.17 mV for GO-PEG due to the positive charge of *N*-(aminopropyl polyethyleneglycol)carbamyl-distearoylphosphatidyl-ethanolamine 1,2-distearoyl-sn-glycero-3-phosphoethanolamine-*N*-[amino(polyethyleneglycol)] (DSPE-PEG-NH_2_), indicating the successful PEGylation of GO. The zeta potentials further increased toward the positive side to −19.3 ± 0.64 mV and −11.7 ± 0.82 mV for GO-CET, and GO-CET/CPT11, respectively, indicating the successful conjugation of CET onto GO-PEG owing to the slightly positive charge of CET (isoelectric point = 8.5) and CPT-11 [39].

The X-ray diffraction (XRD) patterns of graphene and GO are illustrated in Figure 2b. Graphene showed a diffraction peak at 2θ = 26.5°, which could be indexed to the (001) plane of a cubic cell (JCPDS no. 75-2078). After oxidation, a new peak corresponding to the (002) plane of GO is formed at 2θ = 11.6°. The interlayer spacing calculated from the Bragg’s equation increased significantly from 0.336 nm for graphene to 0.745 nm for GO, due to the introduction of oxygen-containing functional groups during the oxidation step for exfoliation [40]. Further confirmation of the GO structure was provided by Raman spectroscopy, which is often used for characterizing graphene and its derivatives by studying the disorder and defects in crystal structure. As shown in Figure 2c, the Raman spectrum of graphene displays a single G-band at 1573 cm^−1^. In contrast, the Raman spectrum of GO displays two strong peaks at 1351 and 1604 cm^−1^, corresponding to the D and G-bands, respectively. The G-band is attributed to the first-order scattering of the sp^2^ carbon–carbon bond [41], while the D-band represents the defect sites associated with vacancies and grain boundaries [42]. During the oxygenation of graphene to form sp3 carbon atoms, the G-band in GO will shift to a higher wavenumber. On the other hand, a broadened D-band will form after oxygenation due to the reduced sp^2^ domain size by the creation of vacancies, defects, and distortions in GO. The I_D_/I_G_ of GO was 0.84, which confirms the successful preparation of GO as a starting nanomaterial for drug loading.

The Fourier transform infrared (FTIR) spectra further confirmed the successful preparation of GO-CET (Figure 2d). The FTIR spectrum of GO showed major peaks at 3400 cm^−1^ (OH), 1731 cm^−1^ (C=O), 1640 cm^−1^ (C=C), 1246 cm^−1^ (C–OH), and 1060 cm^−1^ (C–O). For GO-PEG, additional peaks at 1460 and 2900 cm^−1^ (C–H bending and stretching vibrations in CH_2_) as well as 945 cm^−1^ (P–O–C) could be assigned to DSPE-PEG-NH_2_ in PEGylated GO. As for GO-CET, a new peak at 1640 cm^−1^, corresponding to C=N bond formation between the amine groups of DSPE-PEG-NH_2_ in GO-PEG and the aldehyde groups of activated CET (Figure 1a), provides strong evidence of successful CET conjugation through amide bond formation. From chemical analysis, the amount of NH_2_ in GO-PEG, for conjugation with CET, is estimated to be 1.2 × 10^−4^ mmol/g GO from 2,4,6-trinitrobenzene sulfonic acid (TNBSA) assays. Using excess CET for reacting with GO-PEG, we successfully synthesize GO-CET with 0.14 mg CET/mg GO-CET from protein assays. This density of CET, an EGFR antibody, is deemed suitable to be recognized by the overexpressed EGFR moiety on the U87 cancer cell surface for active targeting [43].

### 2.2. Synthesis and Characterization of CPN

**Figure 3:**
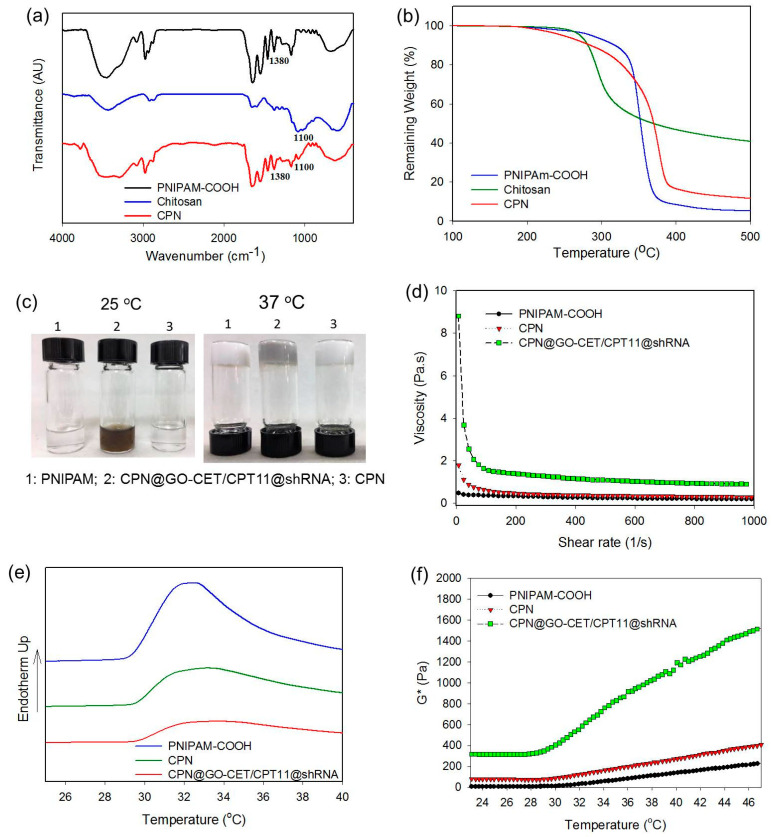
Characterization of PNIPAM-COOH, chitosan, and CPN by Fourier transform infrared (FTIR) spectroscopy (**a**) and thermogravimetric analysis (TGA) (**b**). (**c**) Gross view of sol–gel phase transition of 10% (*w*/*v*) polymer solution from 25 °C (solution) to 37 °C (gel). (**d**) The viscosity of 10% (*w*/*v*) polymer solution at 25 °C. The sol–gel phase transition was analyzed using a differential scanning calorimeter (DSC) (**e**) as well as a rheometer to determine the complex shear modulus (G*) (**f**).

The CPN copolymer was prepared according to Figure 1b and characterized. The zeta potential of PNIPAM-COOH displayed a negative surface potential (−7.85 ± 0.39 mV) due to the dissociation of protons from the –COOH end groups in water. After grafting PNIPAM-COOH to chitosan, the surface potential of CPN turns positive to 6.14 ± 0.76 mV, due to consumption of the negative charges associated with the carboxylate groups during the formation of the neutral amide bonds as well as remnant positive charges associated with protonated primary amine groups in the chitosan backbone. As shown from the FTIR spectra in Figure 3a, PNIPAM–COOH displayed characteristic peaks assigned to amide bonds at 1654 cm^−1^ (C=O) and 1565 cm^−1^ (N–H) as well as C–H peaks assigned to the isopropyl group at 1380 cm^−1^. The characteristic peaks at 1100 cm^−1^, 1565 cm^−1^, and 1654 cm^−1^ in the spectrum of chitosan could be assigned to the C–O stretching of chitosan skeletal ether bonds, N–H bending vibration of the primary amino group, and the C=O stretching vibration. For CPN, we could use the peak corresponding to C–H in the isopropyl groups at 1380 cm^−1^ for the positive identification of PNIPAM in CPN, as characteristic amide bands are overlapping with PNIPAM and chitosan. In addition, the C–O absorption bands of chitosan are clearly observed at 1100 cm^−1^ for identifying the chitosan backbone in CPN. From thermogravimetric analysis (TGA), PNIPAM-COOH showed a peak decomposition temperature at 380 °C with 5.1% residual weight at 500 °C (Figure 3b). By grafting PNIPAM-COOH to chitosan to form CPN, the peak decomposition temperature shifted to 390 °C, together with increased residual weight (11.5%) at 500 °C. Using the difference in the residual weight between PNIPAM-COOH and CPN, the weight percentage of chitosan in CPN could be estimated to be ≈18% considering the residual weight of chitosan (40.7%).

The gross observation of PNIPAM-COOH and CPN polymer solutions indicates both are clear, free-flowing solution and injectable through a syringe needle at 25 °C. At 37 °C, the polymer solution undergoes phase transition from a swollen hydrated state to a shrunken dehydrated state, which forms a rigid hydrogel sticking firmly to the bottom of the inverted vial (Figure 3c). After entrapping GO-CET/CPT11 and shRNA, the CPN polymer solution maintains the same thermo-responsive characteristic as an injectable delivery vehicle for CPT-11 and shRNA, albeit with a change of appearance color from white to black due to the color of GO (Figure 3c). An ideal injectable hydrogel should offer low resistance to flow after shear, thus a shear thinning behavior. This property is expected to allow the hydrogel solution to be injected with little energy consumption once the plunger starts to move, with the viscosity of the hydrogel solution starting to decrease. In order to determine the hydrogel resistance to flow through a syringe, we studied the effect of shear rate on the viscosity of different polymer solutions at 25 °C. As shown in Figure 3d, PNIPAM-COOH, CPN, and CPN@GO-CET/CPT11@shRNA displayed the desirable shear thinning behavior with decreasing viscosity with increasing shear rate, endorsing the applicability of CPN as an injectable hydrogel vehicle for GO-CET/CPT11 and shRNA delivery.

The phase transition behavior of hydrogel was further subject to differential scanning calorimetry (DSC) analysis. As shown from Figure 3e, the sol–gel phase transition starts at 29.5, 29.6, and 29.6 °C for PNIPAM-COOH, CPN, and CPN@GO-CET/CPT11@shRNA, respectively, with the endothermic peak temperatures at 32.3, 33.0, and 33.3 °C. The endothermic enthalpy change decreased from 2.24 to 1.94 J/g after grafting PNIPAM to chitosan, which further decreased to 1.59 J/g after blending with GO-CET/CPT11 and shRNA. This shift in enthalpy change indicates grafting PNIPAM to chitosan, or the entrapment of GO and shRNA in CPN may facilitate gel formation. To evaluate the temperature-responsive change of hydrogel strength, the complex shear modulus (G*) at different temperatures was determined (Figure 3f). The rise of G* values for all samples occurs at around 30 °C, which could be correlated with the sol-to-gel phase transition characteristic of PNIPAM above the lower critical solution temperature. Furthermore, the G* were in the order of CPN@GO-CET/CPT11@shRNA >> CPN > PNIPAM, especially after gel formation, indicating the much improved mechanical property of CPN hydrogel. The significantly enhanced mechanical strength of CPN in the presence of GO and shRNA, which is beneficial for maintaining the shape of the implanted composite hydrogel after intratumoral delivery, could be ascribed to the electrostatic interaction between the positively charged CPN and the negatively charged GO-CET/CPT11 and shRNA. Taken together, encapsulating GO-CET/CPT11 and shRNA in CPN not only retains the thermo-responsive natures of CPN for the injectable delivery of therapeutics, it also greatly strengthens the mechanical property of CPN@GO-CET/CPT11@shRNA composite hydrogel formed in situ.

### 2.3. Loading and Release of CPT-11

**Figure 4:**
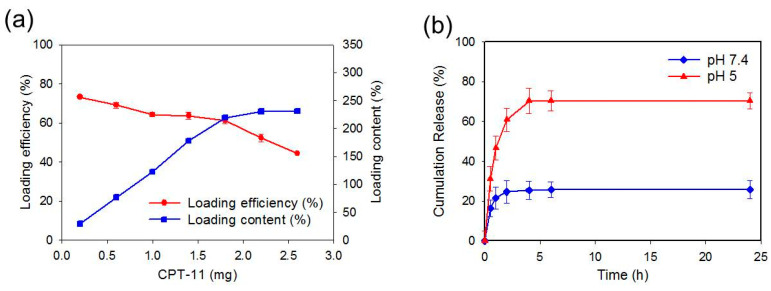
(**a**) Drug loading efficiency (weight percentage of initial CPT-11 loaded on GO-CET) and drug loading content (weight of CPT-11 loaded per unit weight of GO-CET) when a drug solution with different amounts of CPT-11 was reacted with 0.5 mg GO. (**b**) CPT-11 release from GO-CET/CPT11 at pH 7.4 and pH 5 in PBS (37 °C).

The loading efficiency and loading content of CPT-11 on GO-CET was reported in Figure 4a, when different amounts of CPT-11 was mixed with 0.5 mg of GO-CET. At higher CPT-11 concentrations for drug loading, the drug loading content (weight of CPT-11 loaded per unit weight of GO-CET) increased from 29.3% to 231%. On the contrary, the loading efficiency (weight percentage of initial CPT-11 loaded on GO-CET) decreased from 73.2% to 44.4%. As space available for CPT-11 binding on GO-CET is limited, the drug loading content increases with drug concentration up to 1.8 mg, after which it shows saturation by approaching the maximum loading capacity. For drug loading efficiency, the trend shown from the drug loading content gives a somewhat flat loading efficiency curve initially, followed by a rapid decrease of loading efficiency after 1.8 mg CPT-11 (Figure 4a). Thus, a dramatic increase in drug loading content led to a detrimental decrease in loading efficiency. To obtain a suitable formulation, an ideal tradeoff between the loading content and loading efficiency should be taken into consideration. Taken together, missing CPT-11 with 0.5 mg GO-CET was deemed the best formulation, which provided 220% loading content and 61.3% loading efficiency. This preparation was used in all studies herewith.

Since the release of CPT-11 from GO-CET/CPT11 is preferable after endocytosis for maximum anticancer efficacy, a pH-dependent drug release behavior for GO-CET should be confirmed, even though GO has been widely reported to be endowed with such characteristics for chemotherapy drugs [8]. The release of drug from GO-CET/CPT11 was evaluated from the cumulative percentage of CPT-11 released at 37 °C in pH 7.4 and pH 5 phosphate-buffered saline (PBS) to simulate the physiological condition and the endosomal environment, respectively. As shown in Figure 4b, a burst release of CPT-11 from GO-CET/CPT11 was observed to be pH-dependent, where the percentage of CPT-11 released at pH 5 (70.4%) in 96 h is 2.7-fold that at pH 7.4 (25.8%). The adsorption of CPT-11 to GO-CET involves hydrogen bonding between –COOH of GO-CET and –OH of CPT-11. As hydrogen bond formation is pH-dependent, more protons will compete with functional groups responsible for hydrogen bonding at pH 5 than at pH 7.4. This can weaken the interactions between CPT-11 and GO-CET and lead to substantially more released CPT-11 under acidic condition. This pH-sensitive CPT-11 release behavior from GO-CET will facilitate the prompt and enhanced drug release in the acidic endosomal environment, which will cause specific cell killing by poisoning topoisomerase in cancer cells after the endocytosis of released GO-CET/CPT11 from CPN due to shedding of the hydrogel [44].

### 2.4. Degradation of CPN and Release of CPT-11 and shRNA from CPN

**Figure 5:**
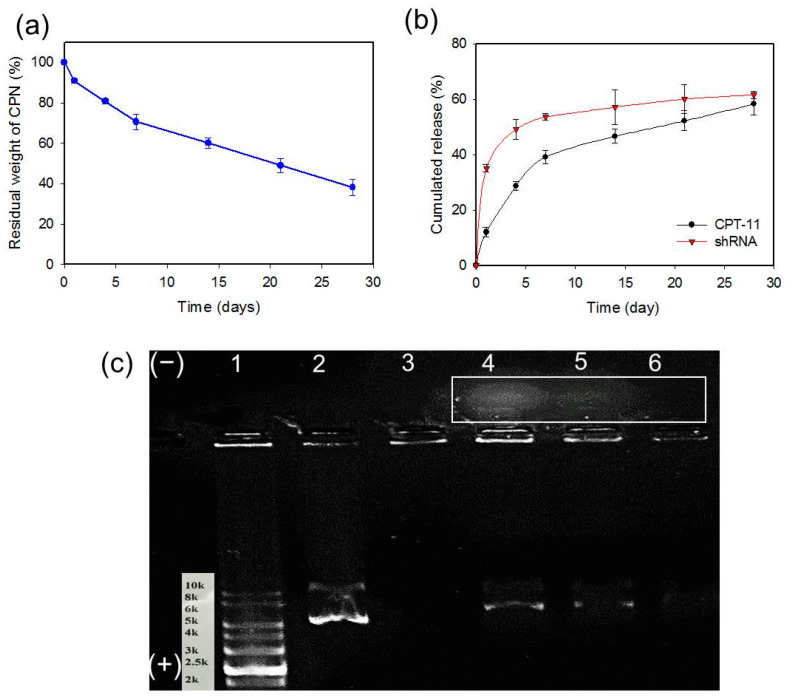
The degradation of CPN in pH 7.4 PBS at 37 °C (**a**) and the release of CPT-11 and SLP2 shRNA from CPN@GO-CET/CPT11@shRNA at pH 7.4 in phosphate-buffered saline (PBS) at 37 °C (**b**). (**c**) The agarose gel electrophoresis analysis of released shRNA. 1: marker; 2: shRNA; 3: release solution of CPN@GO on day 21; 4: release solution of CPN@GO-CET-CPT11@shRNA on day 4; 5: release solution of CPN@GO-CET-CPT11@shRNA an day 14; 6: release solution of CPN@GO-CET-CPT11@shRNA on day 21. The rectangle area indicates a population of shRNA/CPN complex (polyplex) migrating toward the negative electrode detected in the release solution of CPN@GO-CET/CPT11@shRNA.

A desired degradation behavior of hydrogels is important for biomedical applications. Furthermore, as the release of CPT-11 and shRNA CPN is dependent on the degradation of CPN, which leads to the release of GO-CET/CPT11 and shRNA, we studied the degradation (dissolution) of CPN based on the residual weight of hydrogel after incubation in PBS (pH 7.4). CPN showed gradual weight loss due to the dissolution of CPN polymer in PBS with about 62% weight loss in 28 days (Figure 5a). This slow degradation leads to the much improved sustained release of CPT-11 from CPN@GO-CET/CPT11@shRNA up to 28 days, in a slower and controlled manner, compared with GO-CET/CPT11 (burst release in 6 h) (Figure 5b) [45]. Therefore, entrapping GO-CET/CPT11 in CPN provided an effective modulation of the burst release of drug at the extracellular environment (physiological pH). After CPN degradation and the shedding of GO-CET/CPT11 that targets cancer cells through CET/EGFR interaction, copious CPT-11 would be released from GO-CET/CPT11 in the acid endosomal environment of cancer cells due to improved drug release at pH 5 (Figure 4b) to exert maximum therapeutic effect.

A similar sustained release behavior was also observed for shRNA released from CPN@GO-CET/CPT11@shRNA, but with faster kinetics compared to CPT-11 (Figure 5b). A burst release was observed for 7 days followed by sustained release up to 28 days. The release of shRNA also matches the rate of CPN degradation in PBS at pH 7.4 (Figure 5a), suggesting that the dissolution of CPN polymer from the hydrogel leads to the shedding of shRNA from the hydrogel. It should be noted since the release of drug or shRNA from CPN hydrogel was tested in vitro in PBS, we could expect a much higher release rate of CPT-11 and shRNA with the hydrolysis of chitosan by lysozyme in vivo for timely anticancer effects.

The shRNA release was subject to gel electrophoresis studies by gel retardation assays of shRNA in the release solution. As shown in Figure 5c, shRNA was detected in the release solution up to 21 days, to be consistent with its release curve in Figure 5b. The migration ability of released shRNA from CPN@GO-CET/CPT11@shRNA toward the positive electrode (+) was similar to that of free shRNA as expected in open-circular, linear, or supercoiled forms. Nonetheless, a population of smear shRNA bands was found migrating toward the negative electrode (−), in contrast to shRNA that migrated toward the positive electrode (+) originating from the negative charge associated with the phosphate groups present. We postulated that this positively charged shRNA complex arises due to polyplex formation when shRNA was complexed with CPN in the release solution. As CPN molecules were entangled with shRNA in the release solution in different ratios, their positive charges eventually moved shRNA/CPN complexes to the negative electrode (−) [46]. Indeed, the negatively charged shRNA is inclined to be wrapped around by the positively charged CPN polymer chains due to electrostatic interaction. As CPN forms a polyplex with shRNA with N/P ratios (the ratio between the protonated amines from the chitosan and the negatively charged phosphate groups from nucleic acid) higher than 1, the negative charge of shRNA will be reversed into a positive one and can change the migration direction during gel electrophoresis [47]. Taken together, by incorporating shRNA within CPN, the injectable hydrogel will provide a preferred milieu for shRNA delivery by using the degraded CPN copolymer in the solution as a favorable nonviral vector for the delivery of plasmid shRNA, which could protect and transport shRNA to the cytoplasm of cancer cells.

### 2.5. Intracellular Uptake

**Figure 6:**
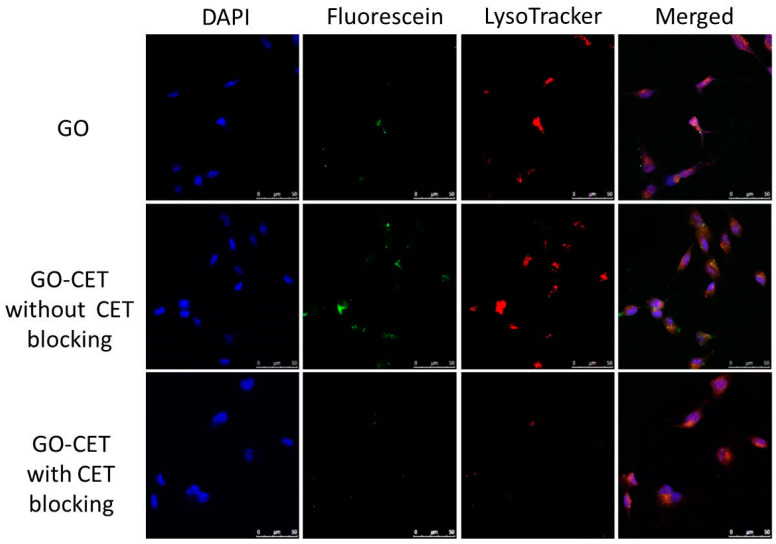
The intracellular uptake of fluorescein-labelled GO and GO-CET (with or without CET blocking) by U87 cells was studied by confocal laser scanning microscopy analysis. The cell nuclei showed blue fluorescence after staining with 4′,6-diamidino-2-phenylindole dihydrochloride (DAPI) and GO (or GO-CET) showed green fluorescence by labeling with fluorescein, and lysosomes showed red fluorescence after staining with LysoTracker. Bar = 50 μm.

The high expression of EFGR on the U87 glioblastoma cell surface is expected to facilitate the active targeting of U87 cells by using CET, an EGFR antibody, as a ligand. This targeting effect could be visualized through the intracellular uptake of fluorescently labeled GO or GO-CET (green fluorescence) under a confocal microscope (Figure 6). The confocal images confirmed that U87 demonstrated a higher intracellular uptake of GO-CET than GO from the intensity of green fluorescence signals. Without EGFR conjugation, GO still exhibited limited fluorescence signals, as nano-sized GO could enter cells mainly through clathrin-mediated endocytosis [48]. The internalization of GO-CET by U87 was restricted when cells were pre-treated with CET, indicating that CET pretreatment will saturate the EGFR on the U87 surface, rendering their inaccessibility to be recognized by GO-CET. To further confirm the process of endocytosis, the U87 cells were also stained for lysosomes with LysoTracker (red fluorescence) in Figure 6. The enclosure of GO-CET within lysosomes after cellular internalization could be clearly identified with merging of green and red fluorescence signals. Taken together, the confocal microscopy results indicate that the intercellular uptake of GO-CET by U87 cells could be augmented via active targeting by conjugated CET.

### 2.6. In Vitro Biocompatibility of GO and Cytotoxicity of CPT-11-Loaded GO

**Figure 7:**
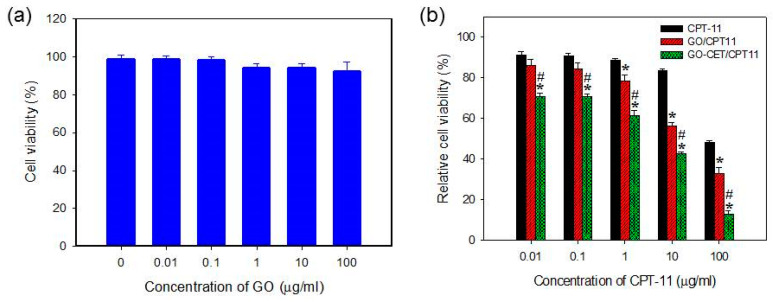
The biocompatibility of GO tested with 3T3 fibroblasts (**a**) and cytotoxicity of CPT-11 toward U87 cancer cells in different formulations (**b**). * *p* < 0.05 compared with CPT-11, ^#^ *p* < 0.05 compared with GO/CPT11.

The in vitro biocompatibility of the GO was evaluated by the 3-(4,5-dimethylthiazol-2-yl)-5-(3-carboxymethoxyphenyl)-2-(4-sulfophenyl)-2H-tetrazolium (MTS) assays using normal cell line (3T3 fibroblasts). From Figure 7a, we could confirm that GO is biocompatible at a concentration up 100 μg/mL in cell culture medium with relative cell viability (compared to culture medium) higher than 90%. Once confirming the cellular internalization characteristic and the safety of the nanocarriers, we sought to study the drug cytotoxic efficacy with free and GO-bound CPT-11. From Figure 7b, GO-CET/CPT11 exerted the most cytotoxic effect toward U87 with the lowest IC_50_ (6.21 μg/mL), in comparison with 32.50 and 94.86 μg/mL for GO/CPT11 and CPT-11, respectively. Undoubtedly, an enhanced cytotoxic effect could be attributed to the CET-mediated active targeting of GO-CET/CPT11 toward the EGFR over-expressed U87 cells. Considering the better performance of GO/CPT11 over CPT-11 at higher drug concentrations, a previous report reported that CPT-11 loaded to GO will lead to higher cytotoxicity in HepG2 and HeLa cell lines due to the limited water solubility of CPT-11, which would be improved by using GO/CPT11 that shows good aqueous solubility [49]. Thus, the difference in cytotoxicity toward U87 cells between CPT-11 and GO/CPT11 was enhanced at higher CPT-11 concentration.

### 2.7. Gene Delivery, Gene Silencing, and Migration Inhibition of U87 Cells with SLP2 shRNA

The RNA interference (RNAi) is a viable strategy for functional gene silencing in tumor cells by inducing function loss of the targeted gene [50]. To mediate the RNAi effect, both small interfering RNA (siRNA) and short hairpin RNA (shRNA) could be used in addition to bi-functional shRNA. With the simplicity of manufacturing and the transient effect, siRNA is most suited for certain medical disorders such as viral injections. In contrast, optimized shRNA constructs will allow for high and sustainable potency at low copy numbers with the endogenous processing mechanism to result in less off-target effects [51]. A nonviral vehicle for the delivery of shRNA is typically a cationic preparation with its positive charge facilitating complexation with negatively charged nucleic acids. Therefore, we use CPN as the vehicle for the delivery of SLP2 shRNA. After shedding from the CPN vehicle, we expect that the CPN polymer will complex with shRNA to form polyplex due to the electrostatic interactions as supported from Figure 5c. The shRNA/CPN complex will bind to the negatively charged cell membrane to promote endocytosis and once endocytosed, CPN’s positive charge could facilitate early escape from the endosome [52]. Once entering the cytoplasm, the shRNA expression vector will be transported into the nucleus for processing into the primary transcripts (pre-shRNAs). The pre-shRNAs will subsequently be transported to the cytoplasm, followed by further processing into mature shRNA to exhibit RNAi function either through translational suppression or mRNA degradation [53].

**Figure 8:**
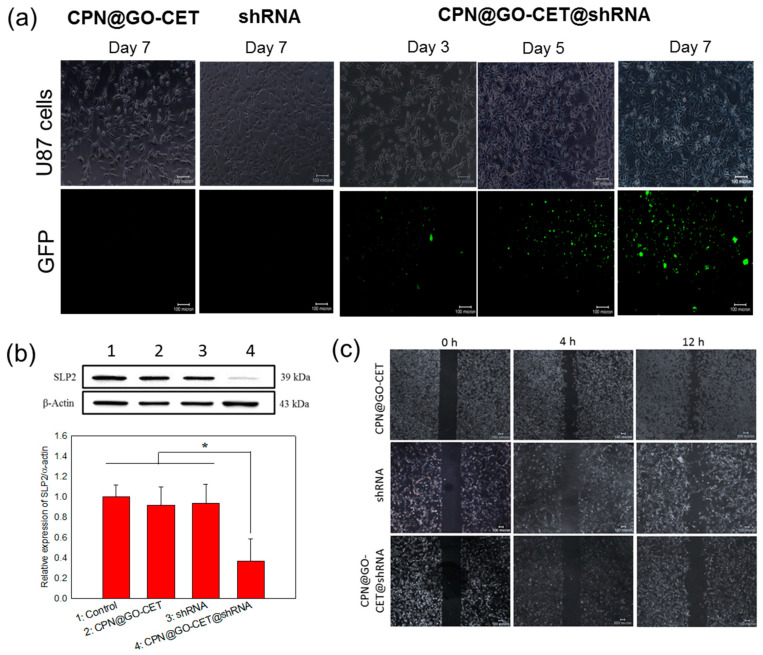
Transfections of U87 cancer cells with CPN@GO-CET@shRNA could knockdown SLP2 gene expression and inhibit cancer cell migration. (**a**) The transfection efficiency was determined from green fluorescent protein (GFP) expression with the fluorescence images shown below the corresponding bright-field images (bar = 100 μm). The effects of SLP2 shRNA on SLP2 expression level in U87 cells were examined by Western blot analysis using β-actin as a loading control (**b**) and wound-healing assays for cell migration ability (bar = 100 μm) (**c**) after U87 cells were transfected with CPN-GO-CET, shRNA and CPN@GO-CET@shRNA for 5 days. * *p* < 0.05.

To confirm that the SLP2 shRNA could be inserted into genes of targeted cancer cells to exert its RNAi function, we proceed to identify the expression of the green fluorescent protein (GFP) in U87 cells after being transfected with different formulations of shRNA by taking advantage of the reporter GFP gene encoded in SLP2 shRNA plasmids. As shown in Figure 8a, CPN@GO-CET@shRNA exhibited much higher transfection efficiency compared to free shRNA, which shows a minimum expression of GFP protein similar to the vehicle without shRNA (CPN@GO-CET), indicating that SLP2 shRNA plasmid could easily enter the negatively charged cellular membranes after being entrapped in and released from the positively charged CPN. This endorsed our intended use of the chitosan-based hydrogel in gene delivery with chitosan reported to be a desirable nonviral vector with advantages such as low toxicity, low immunogenicity, and excellent biocompatibility [54]. Additionally, there is a continuous time-dependent increase of GFP expression (Figure 8a), coinciding with the sustained release of SLP2 shRNA from the CPN hydrogel (Figure 5b). By comparing with the transfection with shRNA where a minimum expression of GFP was found, we demonstrate that the inefficient gene transfection using free SLP2 shRNA could be drastically improved, possibly by the formation of polyplex between shRNA and CPN before entering cell cytoplasm (Figure 5c) [55].

The confirmation of successful knockdown of the SLP2 gene was undertaken by Western blot analysis of SLP2 protein expression in transfected U87 cells. As shown in Figure 8b, consistent with GFP protein expression, the Western blot analysis clearly demonstrated that SLP2 depletion only occurred when U87 cells were transfected with CPN@GO-CET@shRNA, which shows significantly lower SLP2 protein expression than other groups. Indeed, the CPN@GO-CET@SLP2shRNA treatment substantially reduced the level of SLP2 protein to 35% compared to cells transfected with free shRNA and the vehicle (CPN@GO-CET). A previous study shows the inhibition of cell proliferation and mobility and some alteration of cell cycle by knockdown of the SLP2 gene, but without a noticeable change of cell apoptosis rate [56]. To confirm that the depletion of SLP2 will lead to such effects, we used the wound-healing assay, which is an integrated process of cell migration and proliferation, to examine the migration ability of transfected U87 cells. The SLP2-depleted U87 cells and control cells were detached from the well surface 5 days post-transfection. After seeding into new 24-well plates with equal cell numbers, the cells were subjected to scratch wounding on the next day after the formation of a confluent monolayer. As shown in Figure 8c, the migration of U87 was inhibited due to the knockdown of the SLP2 gene. Although the cells in shRNA and CPN@GO-CET groups migrated rapidly into the wound area to cause higher wound closure within 12 h, a significant wound closure inhibition was observed in the SLP-2 depleted cells. The recovered wound areas compared with the initial wound area are 16.7 ± 1.3% and 30.7 ± 2.0% for CPN@GO-CET@shRNA at 4 and 12 h, respectively. These values are significantly less than those for CPN@GO-CET (47.5 ± 2.3% at 4 h and 59.7 ± 2.6% at 12 h) and shRNA (45.6 ± 1.2% at 4 h and 57.9 ± 3.3% at 12 h), which showed no significance between them.

### 2.8. Cell Cytotoxicity and Apoptosis of CPT-11-Loaded GO in CPN

**Figure 9:**
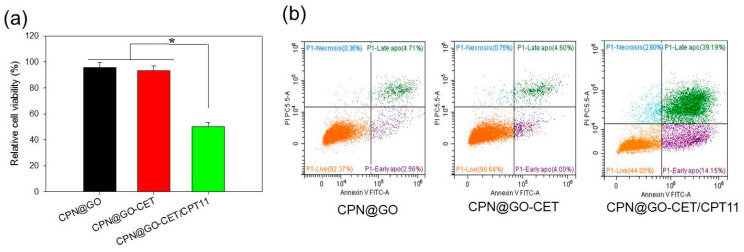
Cell cytotoxicity and cell apoptosis induced by different CPN formulation using U87 cells by 3-(4,5-dimethylthiazol-2-yl)-5-(3-carboxymethoxyphenyl)-2-(4-sulfophenyl)-2H-tetrazolium (MTS) assays (**a**) and flow cytometry (**b**). * *p* < 0.05.

As a final confirmation of the enhanced cytotoxicity of CPN@GO-CET/CPT11 toward U87 cancer cells, we studied cell cytotoxicity by MTS assays and cell apoptosis by flow cytometry in comparison with the vehicle (CPN@GO and CPN@GO-CET). As shown in Figure 9a, the relative cell viability of CPT-11 loaded CPN (CPN@GO-CET/CPT11) is less than half of that in CPT11-free vehicle (CPN@GO and CPN@GO-CET). The less than 5% cell death in CPN@GO and CPN@GO-CET groups also endorse the biocompatibility of CPN.

To further assess the cytotoxic effect of CPN@GO-CET/CPT11, we preformed flow cytometry analysis by staining the cells with Annexin V/propidium iodide (PI) to quantitatively determine the distribution of live, apoptotic, and necrotic cells from the change of cell membrane permeability and integrity (Figure 9b). U87 were co-cultured with CPN@GO-CET and CPN@GO-CET/CPT11 for 48h. The CPN@GO group showed the cell survival and apoptosis rate to be 92.37% and 7.27%, respectively. For CPN@GO-CET, a comparable cell survival (90.64%) and apoptosis rate (8.69%) was observed, supporting the safety and biocompatibility of CPN and GO as before. The slightly higher apoptosis rate in CPN@GO-CET could be attributed to the fact that CET may cause cytotoxicity to EGFR-expressing cancer cells, as CET is a U.S. Food and Drug Administration (FDA)-approved drug for treating colon and head/neck cancers [57]. However, treatment with CPN@GO-CET/CPT11 revealed the most pronounced decrease of cell survival (44.05%) and the increase of apoptotic rate (53.34%), without a significant change in the ratio of necrotic cells, to be consistent with the anti-tumor mechanism of CPT-11. Taken together, in accordance with the anti-tumor mechanism of CPT-11, our data supported that CPN@GO-CET/CPT11 will lead to enhanced U87 cancer cell killing by the induction of apoptosis [58].

### 2.9. Degradation and Biocompatibility of CPN@GO In Vivo

**Figure 10:**
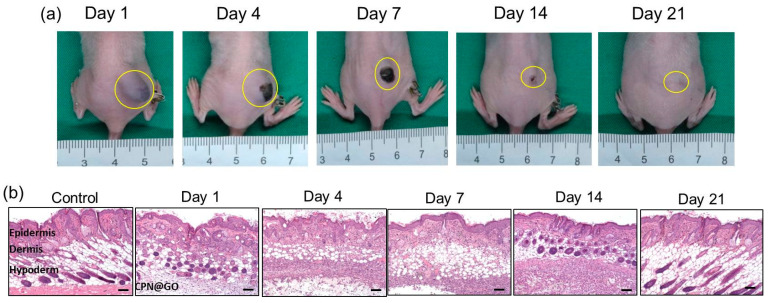
The in vivo degradation of CPN@GO hydrogel after the subcutaneous injection of a polymer solution containing 10% (*w*/*v*) CPN and 0.5% (*w*/*w*) GO to the right flank of a nude mouse. Gross view images were taken at different time points post-implantation (**a**), and the tissues surrounding the hydrogels were dissected and subject to hematoxylin–eosin (H&E) stain (**b**).

As the desired degradation behavior and excellent biocompatibility of hydrogels are crucial for biomedical applications, we started the animal study by investigating the biodegradability and biocompatibility of CPN@GO in vivo (Figure 10a). This was accomplished by injection of 0.2 mL 10% (*w*/*v*) GO-containing CPN hydrogel solution into the subcutaneous pocket in the right flank of a nude mouse to form a rigid gel mass. By continuously monitoring the implanted area, we did not observe any signs when a toxic injectable hydrogel is tested in vivo such as erythematous change around subcutaneous mass, skin necrosis, or even pus formation (Figure 10a) [59]. The gel mass continued to degrade in the subcutaneous layer after implantation, and complete degradation was found after 3 weeks. That CPN hydrogel degraded faster in vivo than in vitro (Figure 5a), which could be due to several factors, is generally in line with previous studies [60]. There was also no significance difference in mouse body weight between the control group without implantation and the CPN@GO group, suggesting that the implantation of CPN@GO might not have a detrimental effect on the mice.

The in vivo biocompatibility was further tested by histological examination (hematoxylin–eosin (H&E) staining) of the tissue surrounding the injected hydrogel at given time points (Figure 10b). No significant necrosis, edema, hemorrhaging, and hyperemia were noted in all tissue sections, except for a mild acute inflammation with enhanced neutrophils in tissues 14 days post-injection. Nevertheless, the inflammation was reduced steadily as the hydrogel degraded completely on day 21, demonstrating an acceptable biocompatibility of CPN hydrogel in vivo.

### 2.10. Anticancer Efficacy in Xenograft Nude Mice Animal Model

**Figure 11:**
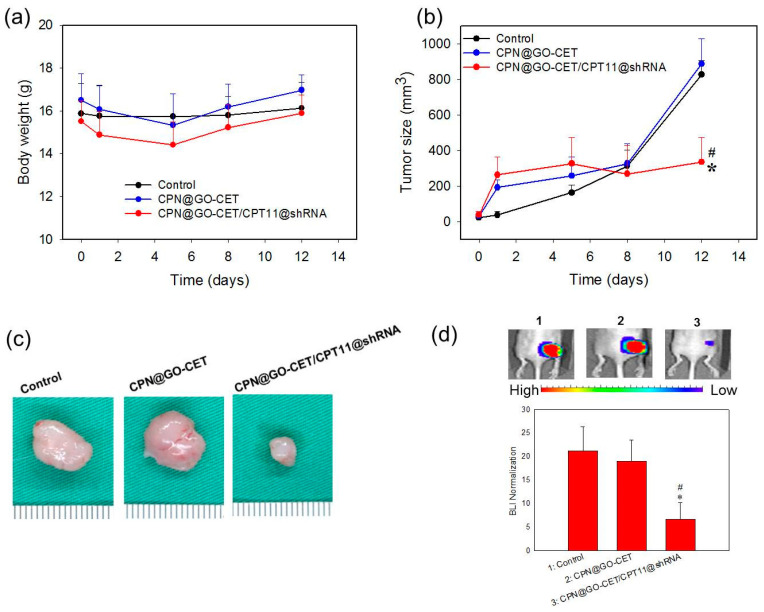
The anti-tumor activity induced by the intratumoral delivery of CPT-11 (40 mg/kg) and SLP2 shRNA (3.33 mg/kg) with CPN to U87 tumor bearing nude mice. The treatment was initiated on day 0 and the tumor size was measured on days 1, 5, 8 and 12 post-treatment. The body weight (**a**) and tumor size (**b**) at different times points were determined for three treatment groups including PBS (control), CPN@GO-CET (vehicle), and CPN@GO-CET/CPT11@shRNA (*n* = 6, mean ± SD). (**c**) The gross view of the explanted tumor 12 days after different treatments. (**d**) Representative bioluminescence imaging (BLI) obtained by in vivo imaging system (IVIS) and the BLI normalization on day 12 (*n* = 6, mean ± SD). * *p* < 0.05 compared with control, ^#^ *p* < 0.05 compared with CPN@GO-CET.

The CPN hydrogels containing SLP2 shRNA and GO-CET/CPT-11 were studied in a xenograft tumor model in mice to evaluate the in vivo combinatory anti-tumor efficacy. For this purpose, BALB/c mice bearing U87 tumors in the right flank area were divided into three groups (*n* = 6 in each group) and subject to intratumoral administration of 50 μL of PBS (control), 10% (*w*/*v*) hydrogel solution (CPN@GO-CET), or 10% (*w*/*v*) CPT-11 and shRNA co-encapsulated hydrogel solution (CPN@GO-CET/CPT11@shRNA). The body weight of mice and tumor size were continuously monitored up to 12 days post-treatment. Although initial weight loss was noted for the hydrogel groups for up to 5 days, no obvious body weight loss was observed at the end of observation period, demonstrating minimum systemic toxicity though the localized injection of hydrogels with or without GO-CET/CPT-11 and shRNA (Figure 11a). Considering the change of tumor size shown in Figure 11b, mice in hydrogel groups (CPN@GO-CET or CPN@GO-CET/CPT11@shRNA) started showing an abrupt increase of tumor size at the first observation period after treatment (day 1) compared with the control, which could be ascribed to the volume occupied by the intratumorally injected hydrogel mass (Figure 10a). Most importantly, the tumor volume increased rapidly during the time series of observation beyond day 1 in the control and CPN@GO-CET groups, in contrast to the CPN@GO-CET/CPT11@shRNA group, in which the tumor size was maintained at nearly a constant value close to that on day 1 to the end of the observation period. On the final observation day (day 12), the tumor volume in the CPN@GO-CET/CPT11@shRNA treated group (335.3 ± 139.1 mm^3^) was significantly less than that in control (826.2 ± 80.9 mm^3^) and CPN-GO-CET groups (886.7 ± 140.5 mm^3^), which showed no significant difference between them. This was also supported from the gross observation of the explanted tumor mass on day 12 in Figure 11c. Taken together, we can conclude that CPN@GO-CET possesses neither anti-tumor efficacy nor systemic toxicity to the host after injection. Nonetheless, treatment with hydrogels loaded with GO-CET/CPT-11 and shRNA displayed a remarkably anti-tumor efficacy, whose mechanism is fully supported from the in vitro experiments. The sustained and controlled release of CPT-11 and SLP2 shRNA from the hydrogel (Figure 5b) will lead to the targeted delivery of CPT-11 (Figure 6) and efficient transfection of U87 (Figure 8a), which results in cell apoptosis by CPT-11 released in the endosomes (Figure 9b) as well as reduced SLP2 protein expression (Figure 8b) and inhibited cell migration (Figure 8c) associated with gene silencing by RNAi.

Bioluminescence imaging (BLI) through an in vivo imaging system (IVIS) has been suggested to be a gold standard for evaluating the preclinical efficacy of drug candidates in brain tumor therapy [61]. On the other hand, as the tumor volume might be influenced by injected hydrogel, we sought to confirm the anti-tumor efficacy results from tumor volume measurement with BLI using IVIS. As shown from Figure 11d, the remarkable anti-tumor efficacy of CPN@GO-CET11/CPT11@shRNA treatment manifests itself from the representative IVIS images of tumor-bearing nude mice on day 12. The value of BLI after normalization was calculated after standardization of the bioluminescent signal intensity on day 12 with that on day 0 when the treatment started. The BLI analysis clearly indicated that the CPN@GO-CET/CPT11@shRNA group showed remarkable treatment benefits over control and CPN@GO-CET with a significant reduction of normalized BLI signal intensity, echoing what has been observed from tumor volume measurements.

**Figure 12:**
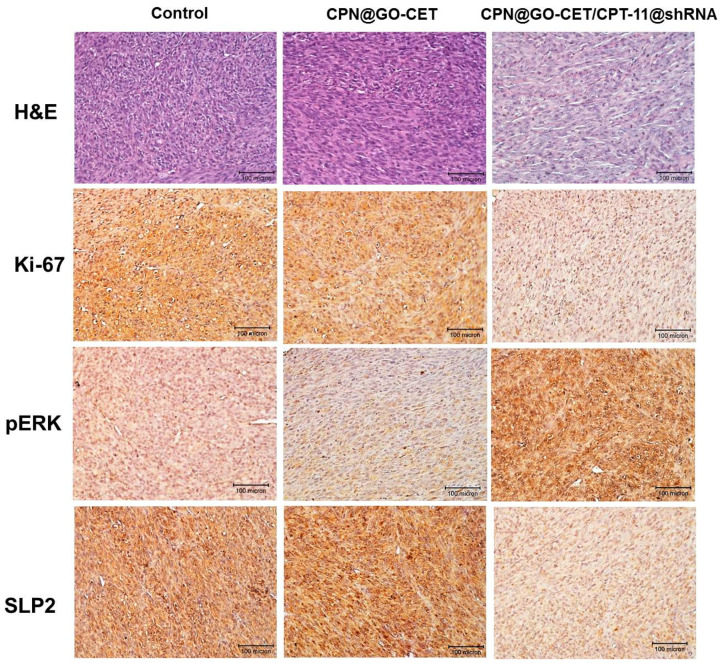
The H&E stain and immunohistochemistry (IHC) analysis of Ki-67, phosphorylated extracellular signal-regulated kinases (pERK), and SLP2 of U87 tumors in control group and groups treated with CPN-GO-CET or CPN@GO-CET/CPT11@SLP2shRNA (bar = 100 μm).

At day 12, the harvested tumors were subject to histological analysis. As shown in Figure 12, the H&E staining indicates the substantial and mild necrosis of tumor tissue in the CPN-GO-CET/CPT11@shRNA group. However, cell growth was noticed in the control and CPN-GO-CET groups. Indeed, there was no evidence of necrosis in the H&E staining slides for tumor samples in the control and CPN-GO-CET group, whereas significantly more necrosis regions were found in tumors treated with CPN@GO-CET/CPT11@shRNA with a more obvious cavitation phenomenon in coagulative necrosis. These results demonstrated that the intratumoral delivery of CPN@GO-CET/CPT11@shRNA enhanced the anti-tumor efficacy, suggesting it to be an excellent treatment for cancer therapy. Improved tumor therapy should also inhibit the proliferation of cancer cells. The immunohistochemistry (IHC) analysis by staining tumor sections with cell proliferation marker Ki-67 antibody showed high immunoreactivity for control and CPN@GO-CET groups due to the active proliferation of tumor cells in tumor samples retrieved from animals in these groups. In comparison, the tumor sample retrieved from animals subject to CPN@GO-CET/CPT11@shRNA treatment displayed weak Ki-67 immunoreactivity with minimum Ki-67 expression due to the apoptosis of U87 cancer cells (Figure 12). These results could be further supported by staining the apoptosis biomarker phosphorylated extracellular signal-regulated kinases (pERK) protein in the tumor tissue (Figure 12), which showed an intense expression of pERK protein only in the tumor tissue of CPN@GO-CET/CPT11@shRNA-treated mice. The cancer cells also showed much less SLP2 protein expression after gene knockdown by the efficient delivery of SLP2 shRNA using CPN to confirm successful RNAi therapeutics in vivo (Figure 12).

## 3. Materials and Methods

### 3.1. Materials

Graphene powder (N002) and *N*-(aminopropyl polyethyleneglycol)carbamyl-distearoylphosphatidyl-ethanolamine 1,2-distearoyl-sn-glycero-3-phosphoethanolamine-*N*-[amino(polyethyleneglycol)] (DSPE-PEG-NH_2_) (PEG chain molecular weight = 2000) were obtained from Angstron Materials Inc. (Dayton, OH, USA) and NOF Co. (White Plains, NY, USA), respectively. The SLP2 shRNA expression vector was constructed and purchased from BIOTOOLS Co. Ltd. (Taipei, Taiwan). 1-(3-dimethylaminopropyl)-3-ethylcarbodiimide hydrochloride (EDC) and *N*-hydroxysuccinimide (NHS) were purchased from Acros Organics (Geel, Belgium). Cetuximab (CET) was purchased from Merck (Darmstadt, Germany). Chitosan (deacetylation degree = 98%, molecular weight = 1.5 × 10^5^ Da), *N*-isopropylacrylamide (NIPAM), azobisisobutyronitrile (AIBN), mercaptoacetic acid (MAA), 2,4,6-trinitrobenzene sulfonic acid (TNBS), 5(6)-carboxyfluorescein *N*-hydroxysuccinimide ester (fluorescein-NHS), 4′,6-diamidino-2-phenylindole dihydrochloride (DAPI), CPT-11, and Dulbecco’s Modified Eagle’s Medium (DMEM) were purchased from Sigma-Aldrich (St. Louis, MO, USA). Hyclone fetal bovine serum (FBS) purchased from Cytiva (Marlborough, MA, USA) was used for cell culture. Potassium salt of D-luciferin was obtained from Gold Biotechnology Inc. (Taipei, Taiwan). LysoTracker™ Deep Red for staining lysosomes, Annexin V FITC/PI cell apoptosis kit for flow cytometry, and Pierce™ BCA protein assay kit were acquired from Thermo Fisher Scientific (Waltham, MA, USA). The CellTiter 96^®^ AQueous One Solution Cell Proliferation Assay (MTS) kit for determination of the number of viable cells was provided by Promega Co. (Madison, WI, USA).

### 3.2. Preparation of GO, GO-PEG, GO-CET, and Fluorescently Labeled GO

Graphene nano-platelet was subject to modification following a modified Hummers method [62]. One gram of graphene powder was added to 23 mL 98% H_2_SO_4_ and stirred on a magnetic stirrer for 12 h, followed by adding 3 g KMnO_4_ with continuous stirring for 30 min at 20 °C, 30 min stirring at 40 °C, and 45 min stirring at 65–80 °C. Forty-six milliliters of distilled deionized water (ddH_2_O) was added to the mixture and stirred at 95 °C for 30 min. After cooling for 1 h at room temperature, 140 mL ddH_2_O and 10 mL H_2_O_2_ solution (30%) were added, followed by reacting at 40 °C for 5 min. After the reaction, GO was washed with 5% HCl solution and centrifuged at 5000× *g* to remove the supernatant. This washing process was continued three times, followed by washing with ddH_2_O three times and dialyzed against ddH_2_O until reaching a neutral pH value. The GO was subject to ultrasonication at 500 W for 30 min using a probe sonicator (Qsonica Q700, Newtown, CT, USA), followed by filtering with a 0.2 μm filter to obtain GO nano-platelet for PEGylation. The PEGylation of GO was achieved by modifying the surface of GO with DSPE-PEG-NH_2_ through the hydrophobic interaction of heptadecyl end groups of DSPE-PEG-NH_2_ with GO_._ Briefly, 1 mL GO solution prepared in pH 7.4 phosphate-buffered saline (PBS) containing 0.5 mg GO was sonicated for 20 min at 100 W, followed by adding 1.5 mg DSPE-PEG-NH_2_ and sonicating for 60 min at 30 W. After centrifugation at 25,000× *g* for 30 min to remove the supernatant containing unreacted PEG, 1 mL pH 7.4 PBS was added to obtain PEGylated GO (GO-PEG). The amount of DSPE-PEG-NH_2_ coated on the GO surface was determined by quantification of its end amine groups using the TNBSA method [63]. This method offers a simple method to determine primary amines by reacting TNBSA with the amine groups to form a chromogenic derivative. By measuring the solution absorbance at 335 nm, the quantification of amines could be achieved by using a standard curve constructed from the amine-containing amino acid glycine.

To conjugate CET onto GO-PEG, amine groups on the GO-PEG surface were reacted with activated CET after oxidation of the carbohydrate moieties in CET (an EGFR antibody) to aldehydes [39]. For this purpose, a 0.1 mg/mL CET solution was prepared in 1 mL ddH_2_O, which was followed by adding 0.2 mL of 0.1 M NaIO_4_ dropwise to open the saccharide rings between vicinal diols and leaving two aldehyde groups for spontaneous reaction with the amine groups on GO-PEG. The solution was purified by passing through a PV-10 desalting column (GE Healthcare, Chicago, IL, USA)) using sodium acetate solution (pH 4.3) as elution buffer and sodium carbonate solution (pH 9.5) as stopping buffer to obtain purified CET-CHO. The purified CET-CHO (40 μL) was reacted with GO-PEG (0.5 mg/mL) in 1 mL sodium carbonate buffer (pH 9.5) for 1 h, which was followed by mixing with 50 μL 0.4 mg/mL NaBH_4_ as a reducing agent to stabilize the covalent bonds. After centrifugation at 40,000× *g* for 30 min, GO-CET was recovered in the precipitate and washed with pH 7.4 PBS twice. The concentration of CET in the collected supernatant was quantified using the BCA protein assay kit to calculate the amount of CET in GO-CET by mass balance. To visualize the intracellular uptake of GO-CET, fluorescently labeled GO or GO-CET was prepared by separately reacting them with fluorescein-NHS for 1 h at room temperature, during which the NHS groups in fluorescein-NHS will spontaneously react with amine groups in GO or GO-CET. After blocking the unreacted amine groups with 1 M glycine, the fluorescently labeled GO or GO-CET was recovered by centrifugation at 40,000× *g* for 30 min.

### 3.3. Characterization of GO-Based Nanocarriers

The particles size, polydispersity (PDI), and zeta potential of nanocarriers were determined by dynamic light scattering (DLS) using a Zetasizer (Nano ZA 90, Malvern Instruments, Malvern, UK). A 0.1 mg/mL particle suspension prepared in distilled water (DI water) was used for measurements. The morphology and size of the particles were observed by transmission electron microscopy (JEOL JEM-2000 EX II, Tokyo, Japan) at 100 kV. Before the observation, the particles was diluted to 0.01 mg/mL in DI water and then dropped onto a 200 mesh carbon-coated copper grid, followed by drying at 25 °C for one day. The Fourier transform infrared (FTIR) spectra were recorded on a Horiba FT-730 FTIR spectrometer (Horiba Ltd., Tokyo, Japan) by mixing samples with KBr powder, and scanned from 1000 to 4000 cm^−1^ at 2.5 mm/s. The crystal structures of samples were analyzed by X-ray diffraction (XRD) and Raman spectra. For XRD analysis, a D2 Phaser X-ray powder diffractometer (Bruker, Billerica, MA, USA) was used by scanning dried power in the 2θ range from 5° to 50°. The step size was 0.02° per second. The phases were compared with the JCPDS database for identification. Raman analysis was recorded between 1100 and 2000 cm^−1^ by a Raman spectrometer (UniD2G, UniNanoTech Inc. Co., Seoul, Korea) with 532 nm laser excitation at 25 mW.

### 3.4. Synthesis of CPN Copolymer

PNIPAM end-capped with a carboxyl group (PNIPAM-COOH) was prepared in benzene by free radical polymerization between NIPAM monomers and MAA using AIBN as an initiator as described before [64]. After the polymerization of NIPAM with MAA under nitrogen atmosphere at 60 °C for 24 h, benzene was removed, and the precipitated polymer was dissolved in acetone and re-precipitated in diethyl ether. This step was repeated 3 times for the purification of synthesized polymer, and the final polymer precipitate was dissolved in water and dialyzed for a week at 4 °C to remove residual reagents and NIPAM. The purified PNIPAM-COOH was lyophilized for storage at room temperature. The CPN copolymer was synthesized by reacting 0.25 g chitosan with 5 g PNIPAM-COOH in 50 mL 2-(N-morpholino)ethanesulfonic acid (MES) buffer (0.1 M, pH 5) containing 0.458 g EDC and 1.375 g NHS for 12 h at 25 °C and purified by thermoprecipitation at 50 °C as detailed in our previous study [36].

### 3.5. Characterization of Hydrogel

The rheological properties of hydrogel were determined using a Discovery HR10 rheometer (TA Instruments, New Castle, DE, USA) with 10% (*w*/*v*) PNIPAM-COOH or CPN samples. The viscosity was determined as a function of shear rate at 25 °C using a 60 mm cone-and-plate from 0 to 1000 s^−1^ in 600 s. The complex shear modulus (G*) was determined at different temperatures from 20 to 50 °C using a 60 mm cone-and-plate at 1 Pa shear stress and 5 Hz frequency. Thermogravimetric analysis (TGA) was conducted with a Q50 TGA (TA Instruments, New Castle, DE, USA). Eight milligrams of powder sample were heated under nitrogen atmosphere from room temperature to 500 °C at a 10 °C/min heating rate. For differential scanning calorimetry (DSC) analysis, 10% (*w*/*v*) CPN hydrogel was put into a DSC aluminum pan and subjected to analysis under 50 mL/min nitrogen using a Q20 DSC (TA Instruments, New Castle, DE, USA) with 5 °C/min heating and cooling rate from 25 to 40 °C.

For in vitro hydrogel degradation, 1 mL of 10% (*w*/*v*) CPN polymer solution containing 0.5 mg GO was prepared by stirring at 4 °C for 1 day. Two hundred microliters of solution prepared above were placed in Millicell cell culture inserts (12 μm pore size) and fitted inside a 24-well cell culture plate and incubated at 37 °C. After the formation of CPN@GO hydrogels, 1.8 mL PBS (pH 7.4) was added to the cell culture plate to fully immerse the hydrogel formed in the cell insert. The plate was incubated at 37 °C by shaking at 50 rpm for the degradation (dissolution) of CPN. At predetermined time points, inserts were removed from the plate, freeze dried, and the residual weight of CPN@GO was determined. For in vivo hydrogel degradation and biocompatibility, a 10% (*w*/*v*) CPN@GO polymer solution (0.2 mL) was subcutaneously injected into the right dorsal region of a nude mouse by using a 1 mL syringe fitted with a 25-gauge needle. The size of the formed gel in the subcutaneous layer was observed continuously and photographed at given time points. The skin tissues surrounding the gel mass were surgically removed and histologically stained by hematoxylin–eosin (H&E) for the examination of inflammatory response to the implanted materials.

### 3.6. Drug Loading and Release

The loading of CPT-11 onto GO-CET was studied by mixing 0.5 mg GO-CET with different amounts of CPT-11 in 1 mL PBS (pH 7.4) at 4 °C for 12 h, followed by centrifugation (30,000× *g*, 15 min). The amount of loaded drug was calculated from mass balance by determining the concentration of CPT-11 in the supernatant with high-performance liquid chromatography (HPLC) at 370 nm. An Eclipse XDB-C18 column (250 mm × 4.6 mm) was used with 0.01 M pH 4 phosphate/75% acetonitrile in a 4/6 volume ratio as the mobile phase at a 1 mL/min flow rate. The drug loading efficiency (%) is defined as weight of CPT-11 loaded/weight of CPT-11 added × 100; the drug loading content (%) is defined as weight of CPT-11 loaded/weight of GO added × 100.

For drug release, GO-CET/CPT-11 was placed in 1 mL PBS (pH 5.7 or pH 7.4) at 37 °C and shaken at 120 rpm. Centrifugation (30,000× *g*, 15 min) was used to separate GO-CET/CPT11 at predetermined time points, and the precipitate was re-suspended with 1 mL fresh PBS of the same pH value as before. The cumulative amount of CPT-11 released from GO-CET/CPT11 (%) was determined from CPT-11 concentration in the supernatant using HPLC and is defined as cumulative weight of released CPT-11/weight of loaded CPT-11 × 100 [65].

To determine CPT-11 release from hydrogel, 0.1 g CPN was dissolved in 1 mL solution containing 0.5 mg GO-CET/CPT11 and 60 μg shRNA by stirring at 4 °C for 1 day. Two hundred microliters of solution prepared above were placed in Millicell cell culture inserts (12 μm pore size) fitted inside a 24-well cell culture plate and incubated at 37 °C. After the formation of CPN@GO-CET/CPT11@shRNA hydrogel, 1.8 mL PBS (pH 7.4) was added to the cell culture plate to fully immerse the hydrogel formed in the insert. The plate was incubated at 37 °C by shaking at 50 rpm for release of CPT-11. At predetermined time points, the solution in each well was removed for the determination of CPT-11 concentration by using HPLC at 370 nm to calculate the cumulative drug release.

### 3.7. SLP2 shRNA Loading and Release

An improved SLP2 shRNA plasmid (SLP2-shRNA-PGLV3/GFP) vector that co-expresses green fluorescent protein (GFP) and SLP2 shRNA simultaneously to facilitate the analysis of silencing at the level of individual cells as well as siRNA for the specific silencing of SLP2 gene expression was used in this study. The shRNA plasmids consist of the TOOLSilent shRNA vectors pGLV3/H1/GFP/Puro vector plasmid encoding SLP-specific 19 nucleotide shRNA designed to knockdown gene expression with 63,000 Da molecular weight [66]. A CPN@GO-CET/CPT11@shRNA polymer solution was prepared by mixing GO-CET/CPT11 and SLP2 shRNA in 0.2 mL 10% (*w*/*v*) CPN polymer solution. The polymer solution was placed in Millicell cell culture inserts fitted in a 24-well cell culture plate and incubated at 37 °C for gel formation. To determine SLP2 shRNA release from CPN@GO-CET/CPT11@shRNA, PBS buffer (pH 7.4) was added to each well after gel formation to fully immerse the hydrogel in the insert, and the plate was further incubated at 37 °C by shaking at 50 rpm. At predetermined time points, solution in each well was completely removed for the determination of molar concentration of released shRNA with a NanoDrop 2000c spectrophotometer (Thermo Fisher Scientific, Waltham, MA, USA). The released shRNA was also determined by gel retardation assays using 8% (*w*/*v*) agarose gel electrophoresis by loading 10 μL sample and 2 μL 6× SYBR Green DNA dye (Invitrogen, Madison, WI, USA). After separating at 100 V for 30 min, the gel was subject to chemiluminescence imaging using MultiGel-21 (TopBio, Taipei, Taiwan).

### 3.8. Cell Line and Cell Culture Condition

A U87 human primary glioblastoma cell line was obtained from American Type Culture Collection (ATCC HTB1, Manassas, VA, USA). The U87 cells were genetically modified by lentiviral infection to express firefly luciferase for in vivo bioluminescence imaging. For cell culture, Dulbecco’s Modified Eagle’s Medium (DMEM) supplemented with 10% FBS and 1% penicillin/streptomycin was used by incubating at 37 °C in a humidified 5% CO_2_ incubator.

### 3.9. Intracellular Upake

To monitor the intracellular uptake of nanocarriers, U87 cells were seed in a 24-well cell culture plate at a seeding density of 1 × 10^4^ cell/well. After being cultured for 24 h, fluorescently labeled GO or GO-CET (green fluorescence) were separately added to each well and incubated for another 12 h. Treated cells were washed with PBS three times before being fixed in 4% paraformaldehyde. After treating with 0.5 mL 0.1% Triton X-100 and washing, the fixed cells were stained for lysosomes with LysoTracker (red fluorescence) for 30 min and counter-stained for cell nucleus with DAPI (blue fluorescence) for 10 min. To further confirm the ligand-mediated intracellular uptake of GO-CET by binding to EGFR on the U87 surface, CET blocking was performed by pre-treating U87 cells with CET (1 mg/mL) for 1 h before incubating with GO-CET. The samples were observed by a confocal laser scanning microscope (CLSM, BioRad Radiance MRP2100, Hercules, CA, USA) at an excitation wavelength 577/364/340 nm (red/green/blue) and emission wavelength 590/480/488 nm (red/green/blue).

### 3.10. Biocompatibility In Vitro

To evaluate the biocompatibility of GO, 3T3 fibroblasts were seed in a 96-well cell culture plate at a seeding density of 2.5 × 10^3^ cell/well. After removing the culture medium, the cells were treated with different concentrations of GO in 100 µl cell culture medium and cultured at 37 °C for another 24 h. The relative cell viability (compared to cell culture medium) was determined from MTS assay by measuring the solution absorbance at 490 nm (OD_490_) with a microplate reader. The biocompatibility of CPN, CPN@GO, and CPN@GO-CET was determined similarly with 3T3 fibroblasts by individually placing 0.2 mL hydrogel solution in a Millicell insert. After gel formation at 37 °C, the insert was fitted in a 24-well cell culture plate seeded with cells at 2.5 × 10^3^ cell/well and cultured at 37 °C for 24 h. The relative cell viability (compared to cell culture medium) was determined as before.

### 3.11. Cytotoxicity In Vitro

To determine the cytotoxicity in vitro, U87 cells were seeded in a 96-well cell culture plate at 2.5 × 10^3^ cell/well and cultured for 24 h. After washing with PBS (pH 7.4), the cells were incubated with 100 μL of CPT-11, GO/CPT11, or GO-CET/CPT11 solution prepared in culture medium with different CPT-11 concentrations to determine the IC_50_ (half-maximum inhibitory concentration) value. The relative cell viability was determined from MTS assay after culture for 24 h and normalized with the OD_490_ from cell culture medium (for CPT-11), GO (for GO/CPT11), or GO-CET (for GO-CET/CPT11). To determine the cytotoxicity of CPT-11 released from CPN hydrogel, we performed flow cytometry analysis to determine the apoptosis of U87 cells in the presence of CPN@GO, CPN@GO-CET, or CPN@GO-CET/CPT11. The hydrogel sample was prepared in a Millicell insert at 37 °C as before and tested with 1 × 10^4^ U87 cells/well for 48 h. Cells were detached by adding 1 mL trypsin/ethylenediaminetetraacetic acid (EDTA) (0.1%), and the detached cell suspension was reacted with FITC-Annexin V for 30 min and then with propidium iodide (PI) for flow cytometry analysis using a CytoFLEX flow cytometer (Beckman Coulter, Brea, CA, USA).

### 3.12. Transfection of U87 Cells

The GFP gene fragment encoded within SLP2 shRNA allows for the easy monitoring of the fluorescent signal after U87 cells were transfected under different treatments. To investigate SLP2 shRNA transfection, U87 cells were seed in a 24-well cell culture plate at 5 × 10^3^ cells/well and cultured for 24 h. A 10% (*w*/*v*) CPN polymer solution was used to form CPN@GO-CET or CPN@GO-CET@shRNA (with 12 μg shRNA) in Millicell inserts and fitted inside a 24-well cell culture plate for incubation at 37 °C. Transfection with free shRNA was also carried out by directly incubating cells with shRNA at the same gene dosage. After removing the medium and washing each well with PBS at predetermined time points, the gene delivery efficiency was examined by observing the green fluorescence of expressed GFP under an inverted fluorescence microscope (Olympus IX-71, Tokyo, Japan).

### 3.13. Western Blot Analysis

The knockdown of SLP2 gene was determined form Western blot using U87 cells. U87 cells were seeded in a 6-well cell culture plate at a seeding density of 2 × 10^5^ cell/well for 24 h. Cultured cells were subject to treatment with CPN@GO-CET, CPN@GO-CET, or CPN@GO-CET@shRNA as before for 5 days at 37 °C. After washing with PBS and harvesting with 0.1% trypsin/EDTA, cells were treated with lysis buffer containing a protease inhibitor cocktail for 30 min on ice to extract the total protein. After centrifugation to remove cell debris, the supernatant was recovered, and the protein concentration was determined by the BCA protein assay kit. The protein was heat-denatured at 95 °C for 10 min in sample buffer and an aliquot of cell lysates (≈25 μg total protein/lane) was separated by sodium dodecyl sulfate polyacrylamide gel electrophoresis at 50 V for 30 min and then at 110 V for additional 1 h. The gels were transferred to a polyvinylidene fluoride membrane, blocked with 5% fat-free milk for 1 h for nonspecific binding, and blotted with SLP2 or β-actin antibodies for 12 h at 4 °C. After probing with horseradish peroxidase-conjugated secondary antibody (IgG-HRP) and color development with enhanced chemiluminescence (ECL) Western blotting substrate, the immune complexes were detected using a MultiGel-21 image system. The densitometric analysis of specific bands from Western blot was performed using the ImageJ software.

### 3.14. Xenograft Nude Mice Animal Model

BALB/c nude mice (4−6 weeks old, female) were purchased from BioLASCO (Taipei, Taiwan). All animal experiments were conducted according to protocols that were approved by the Chang Gung University’s Institutional Animal Care and Use Committee (IACUC Approval No.: CGU105-034, 2018-01-21). Using a 25-gauge needle, 200 μL cell culture medium containing 1 × 10^6^ U87 cells was administered subcutaneously to the right flank of a nude mouse. The tumor size was monitored daily using a caliper, and the tumor volume was calculated from length × (width)^2^/2. After 7 days when the tumor size was > 30 mm^3^, the tumor-bearing mice were randomly divided into three groups (*n* = 6 in each group) for treatment.

### 3.15. In Vivo Anti-Tumor Efficacy

Each mouse was subject to intratumoral injection of 50 μL of a tested sample. The study group included the following: 1, PBS (control); 2, CPN@GO-CET (10% CPN, 18.35 mg/kg GO-CET); 3, CPN@GO-CET/CPT11-shRNA (10% CPN, 18.35 mg/kg GO-CET 40 mg/kg CPT-11 and 3.33 mg/kg shRNA). After administration, the tumor size and body weight were monitored on day 1, 5, 8, and 12 post-treatment. On day 12, the mice were anesthetized with 1% isoflurane followed by the injection of 100 μL of D-luciferin solution at a dose of 15 mg luciferin/kg body weight. The bioluminescence imaging (BLI) was performed using a non-invasive in vivo imaging system (IVIS) (Xenogen IVIS-200, Caliper Life Sciences) to determine the peak bioluminescence. The BLI intensity was determined by measuring the total bioluminescent signal intensity in the tumor and BLI normalization was done by dividing the total signal intensity with the total signal intensity when the treatment started (day 0). Tumors were harvested at the end of the observation period, and tumor tissues were fixed immediately in 10% buffered formalin, followed by paraffin embedment and sectioning to 5 µm thickness for hematoxylin and eosin (H&E) staining. As for immunohistochemistry (IHC) analysis, the expression of SLP2, pERK and Ki-67 were detected by incubating tissue slices with rabbit primary antibody against the respective protein for 24 h at 4 °C, followed by incubating with HRP-conjugated anti-rabbit secondary antibody (ImmPRESS^®^ HRP universal antibody, anti-rabbit IgG produced in horse) for 30 min. After color development with ImmPACT^®^ DAB EqV Peroxidase (HRP) Substrate and counterstained with hematoxylin for nucleus, images were taken under an inverted microscope.

### 3.16. Statistical Analysis

All results are presented as the mean ± standard deviation (SD). To compare means of different groups, one-way analysis of variance (ANOVA) analysis was carried out using the SPSS software. Differences were considered to be significant at *p* < 0.05.

## 4. Conclusions

We presented a strategy by the localized, sustained co-delivery of CPT-11 and SLP2 shRNA with CET-conjugated GO entrapped in thermosensitive CPN hydrogel. The formulation retained the thermo-responsive phase-transition characteristic of PNIPAM at body temperature as well as the pH-sensitive drug release behavior of GO. A combination of positively charged CPN and negatively charged nanocarrier led to the controlled drug release and improved mechanical strength of the in situ formed hydrogel. A sustained release behavior up to 28 days was observed for CPT-11 release from drug-loaded hydrogel, while the complex shear modulus increased five times after entrapping GO-CET in the hydrogel. Furthermore, conjugation with the EGFR-specific antibody CET facilitates the cellular internalization of GO. The formulation provided enhanced in vitro anti-tumor efficacy by inducing 53% apoptotic rate in 2 days. The SLP2 gene knockdown led to a 65% reduction of SLP2 protein expression and 50% reduction of cell migratory ability in 5 days. The therapeutic efficacy was demonstrated with a xenograft tumor model in nude mice through the intratumoral injection of CPN@GO-CET/CPT11@shRNA, which showed 40% tumor size compared with the untreated control group after 12 days. Overall, this multifunctional drug/gene delivery platform is suitable for localized application with enhanced cancer selectivity and minimal adverse effects.

## Abbreviations

GO	Graphene oxide
CET	Cetuximab
EGFR	Epidermal growth factor receptor
SLP2	Stomatin-like protein 2
shRNA	Short hairpin RNA
siRNA	Small interfering RNA
CPN	Chitosan-g-poly(N-isopropylacrylamide)
GBM	Glioblastoma multiforme
RNAi	RNA interference
WHO	World Health Organization
NF-κB	Nuclear factor kappa-light-chain-enhancer of activated B cells
PNIPAM	Poly(N-isopropylacrylamide)
DSPE	1,2-Distearoyl-sn-glycero-3-phosphorylethanolamine
PEG	Polyethylene glycol
PNIPAM-COOH	Carboxylic acid-ended poly(N-isopropylacrylamide)
NIPAM	N-isopropylacrylamide
MAA	Mercaptoacetic acid
DLS	Dynamic light scattering
XRD	X-ray diffraction
FTIR	Fourier transform infrared
TNBSA	2,4,6-Trinitrobenzene sulfonic acid
DSC	Differential scanning calorimeter
GFP	Green fluorescent protein
PI	Propidium iodide
BLI	Bioluminescence imaging
IHC	Immunohistochemistry
pERK	Phosphorylated extracellular signal-regulated kinases
EDC	1-(3-Dimethylaminopropyl)-3-ethylcarbodiimide hydrochloride
NHS	N-hydroxysuccinimide
DAPI	4′,6-Diamidino-2-phenylindole dihydrochloride
DMEM	Dulbecco’s Modified Eagle’s Medium
FBS	Fetal bovine serum
MES	2-(N-morpholino)ethanesulfonic acid
TGA	Thermogravimetric analysis
H&E	Hematoxylin–eosin
PBS	Phosphate buffered saline
HPLC	High-performance liquid chromatography
CLSM	Confocal laser scanning microscope
EDTA	Ethylenediaminetetraacetic acid
